# Brodie on Mercury in Syphilis

**Published:** 1844-11

**Authors:** 


*Brodie on the best mode of administering mercury in Syphilis.*—But if you inquire which is the best way of giving mercury in cases of syphilis where the symptoms are not of the very mildest character, I must say that mercurial inunction is infinitely to be preferred to mercury taken by the mouth. Mercurial inunction, however, is dirty, laborious, and troublesome, and it makes the case public to the family in which the man lives. For these reasons it will be objectionable to the patient; but it has this advantage, it is much less liable to gripe and purge, and it cures the disease a great deal better. It does not damage the constitution half so much as mercury taken by the mouth; nay, I will go so far as to say that, except in the very slightest cases, you really cannot depend upon any mercurial treatment effecting a certain cure, or even giving a good chance for it, by any other means than inunction. You may often patch up the disease by giving mercury internally, but it will return again and again and again, and you may cure it again by a good course of mercurial ointment. But especial care must be taken that this is properly applied. If it be left to a patient he will rub it in for five minutes or so, whereas it requires to be rubbed in before the fire for three-quarters of an hour ere it enters; but by and by the friction may be continued for a shorter period. Where the symptoms are not of the mildest character it is desirable that the patient should, if possible, be confined to the house. Mr. Pearson observed, long since, that going into the fresh air would undo the effect of mercury, and I never would be responsible for thoroughly eradicating the disease where the patient is at all exposed to cold, and where he does not lead a most careful and regular life.

Wherever you can in the treatment of syphilis, make the patient take mercury in the form of inunction if possible. It is the best plan to pursue in all cases, although it is not necessary in all cases; but where the symptoms are severe, and a long course is required, it is the safest mode of proceeding.

I will avail myself of this opportunity of stating a class of cases in which you may employ mercurial inunctions with the greatest advantage. Children when born, sometimes labor under syphilis, the father or mother having been affected with it—perhaps the father and not the mother. The child at birth looks thin, and is of small size, and instead of thriving it becomes still thinner. At the end of three weeks it is covered by a nasty scaly eruption; there is a sort of aphthæ in the mouth, and chaps about the lips and anus. I have tried different ways of treating such cases. I have given the child grey powder internally, and given mercury to the wet nurse. But mercury exhibited to a child by the mouth generally gripes and purges, seldom doing any good; and given to the wet nurse it does not answer very well, and certainly is a very cruel practice. The mode in which I have treated such cases for some years past has been this—I have spread mercurial ointment, spread over a flannel roller, and bound it round the child once a day. The child kicks about, and the cuticle being thin the mercury is absorbed. It does not either gripe or purge, nor does it make the gums sore, but cures the disease I have adopted this practice in a great many cases with the most signal success. Very few children recover in whom mercury is given internally, but I have not seen a case where this method of treatment has failed.—*Braithwaite's Retrospect.*

